# Knowledge, attitude, behaviour, and influencing factors of home-based medication safety among community-dwelling older adults with chronic diseases: a cross-sectional study

**DOI:** 10.1186/s12877-023-03966-3

**Published:** 2023-04-28

**Authors:** Yang Yu-ting, Yang Yong-wei, Yao Miao, Ye Qiong, Wu Meng-yu, Lin Ting

**Affiliations:** 1 Nursing Department, the First Affiliated Hospital of Fujian Medical University, Fuzhou, Fujian China; 2 The School of Nursing, Fujian Medical University, Fuzhou, Fujian China; 3 Ningde Municipal Hospital of Ningde Normal University, Ningde, Fujian China; 4 Community Health Service Center of Ninghua Street, Taijiang District, Fuzhou, Fujian China

**Keywords:** Older adults, Knowledge, Attitude, Behaviour, Medication safety

## Abstract

**Background:**

Older adults with chronic diseases require long-term medication. However, due to lack of drug knowledge and hypomnesia, older adults with chronic diseases are prone to adverse drug events and increased medical costs. This study aimed to explore the status and influencing factors of home-based medication safety among community-dwelling older adults with chronic diseases in China to provide a basis for follow-up to conduct targeted health education.

**Methods:**

Overall, 427 community-dwelling older adults with chronic diseases participated in this study. The Knowledge, Attitude, and Behaviour of Medication Safety among Older Adults with Chronic Diseases Questionnaire was used to assess their home-based medication safety. Multivariate linear regression was used to identify the factors influencing knowledge, attitude, and behaviour regarding medication safety.

**Results:**

The average score of home-based medication safety among older adults with chronic diseases was 68.26 ± 8.96, indicating that they had a moderate grasp of medication safety. The scoring rate of each subscale was ranked from high to low as follows: behaviour (84.51%), knowledge (63.33%), and attitude (47.39%). Stepwise linear regression analysis showed that medication safety knowledge, attitudes, and behaviours were significantly associated with higher monthly income, adverse drug events, and taking medicine several times a day (*p* < 0.05). Additional influencing factors included having fewer chronic diseases, being female, higher educational attainment, taking medicines multiple kinds a day, better self-care ability, and non-hospitalisation for chronic illnesses (*p* < 0.05).

**Conclusion:**

Medical staff and community workers should pay attention to the drug safety of older adults with different characteristics and mobilise their enthusiasm for participation to improve their medication self-management ability.

**Trial registration:**

Chinese Clinical Trial Register: ChiCTR2200060987; reg. date: 15/06/2022.

**Supplementary Information:**

The online version contains supplementary material available at 10.1186/s12877-023-03966-3.

## Background

China has a prominent aging population, and the number of older adults with chronic diseases is increasing. The Seventh National Census [1] showed that as of May 2020, there were 264 million people aged 60 years and above in China, accounting for 18.70% of the total population. With the acceleration of the aging population, many Chinese older adults are more likely to suffer from chronic diseases. According to the Healthy China Initiative Action (2019–2030) [2], around 180 million Chinese older adults (75%) suffer from one or more chronic diseases, while 90% receive home-based care [3]. The prevalence of chronic diseases among older adults receiving home-based care is as high as 81.37% [4]. Currently, the primary chronic illnesses affecting the health of Chinese adults aged over 60 years are hypertension, diabetes, and hypercholesterolaemia, with prevalence rates of 58.3%, 19.4%, and 10.5%, respectively [5]. As lifelong diseases with high disability and mortality rates, chronic diseases seriously affect older adults’ independent living function and quality of life [6].

Medication remains the primary method for the treatment and control of chronic diseases in older patients [7]. The treatment of chronic diseases usually involves multiple drugs [8], so medication knowledge is a significant component of rational medication use [9]. However, hypomnesia and lack of drug knowledge [10] among older patients who take drugs at home may lead to poor drug management and compliance [11, 12]. Coupled with a lack of professional supervision and guidance, the probability of adverse drug events in older adults with chronic diseases during home medication is increasing [13]. Patients who take medication for chronic diseases lack knowledge of drug side-effects, which can potentially affect medication compliance and safety and increase the medical burden [14]. In Japan, a study found that cognition of drug effects was crucial for improving medication compliance in older patients with type 2 diabetes. When providing patients with medication information, pharmacists can identify specific drug effects to maintain good medication compliance [15]. In China, health education for older adults with multimorbidity could raise their awareness of irrational drug use and identify problems with medication use and related health issues in advance. However, if older adults are only weakly aware of rational drug use, it is difficult to fully realise the value of medication management to improve their health [16]. Therefore, the medication safety of older adults with chronic diseases has become a major social concern [17]. Medication safety [18, 19] refers to the comprehensive assessment and testing of patients’ genes, conditions, constitution, family genetic history, drug ingredients, accurate selection of drugs, accurate medication (with correct methods, doses, and time), attention to drug contraindications, adverse drug events, and interactions, so as to ensure safe, valid, reasonable, and economic use of medication.

The theory of knowledge, attitude, and practice is a mature model of behavioural change proposed by Mayo and Walling [20]. It emphasises that after receiving information, people will use that information to generate beliefs, which, in turn, will form attitudes and behaviours. Therefore, people should obtain appropriate and rich knowledge to establish correct beliefs, adopt positive attitudes, and form healthy behaviours [21]. This theoretical model has been extensively applied in medical care [22], nursing [23], and health education [24]. Accordingly, we used it to assess the knowledge, attitude, and behaviour of community-dwelling older adults with chronic diseases regarding their medication safety, and analysed the influencing factors. We hope to provide a scientific basis for conducting further targeted health education activities and popularising the knowledge of medication safety among older adults with chronic diseases.

## Methods

### Study site

Fuzhou is in southern China and is the capital of Fujian Province. The city has a total area of 11 968 km^2^ and a built-up area of 416 km^2^. As of 2021, the permanent population is 8.42 million and the urbanisation rate is 73% [25]. The Seventh National Census reveals that Fuzhou has 1 389 989 people aged 60 years and above (16.76% of the population), of which 971 594 are aged 65 years and above (11.72%). Compared to the Sixth National Census in 2010, the proportion of the population aged 60 years and above in Fuzhou has increased by 4.67% points, while the population aged 65 years and above has increased by 3.51% points [26].

### Study design and participants

This cross-sectional survey was conducted from May to August 2022 in Fuzhou. We adopted multistage random cluster sampling to obtain a representative sample. First, we numbered each district in the order of the six districts in Fuzhou shown on the government’s website. Second, we randomly selected one district in Fuzhou using the Research Randomizer website (https://www.randomizer.org/). Third, we randomly selected one street from this district and four residential areas from this street using the same website. The four residential areas were from Ninghua Street in Taijiang District. We invited all older adults who met the inclusion criteria, agreed to participate in the study, and resided in these four residential areas. The inclusion criteria were aged older than 60 years, had lived in the urban residential areas for 6 months or more, had been diagnosed with a chronic disease by a hospital of grade II or above, could express and comprehend simple Chinese characters, and was willing to complete questionnaires. The exclusion criteria were those who had hearing impairments, speech impairments, severe cognitive impairments, mental illnesses, and other severe or terminal diseases. Overall, 434 participants were included in this study.

All participants were informed of the study procedure upon their recruitment. After obtaining written consent, trained interviewers interviewed the participants. Of the sample, 434 participants were invited to answer a standardised questionnaire, and 427 participants completed the study. The response rate was 98.39% (427/434). This study was reviewed and approved by the Ethics Review Committee of Fujian Medical University (No.: 2022/00075).

### Measurements

#### Participants’ demographic characteristics questionnaire

This questionnaire was compiled by the researchers according to the study purpose and relevant literature, and included items on age, sex, education, marriage, residential status, number of children, occupation before retirement, source of income, monthly personal income, medical insurance type, self-care, number of chronic diseases, duration of chronic diseases, and so on (see Supplementary material).

#### Knowledge, attitude, and behaviour of medication safety among older adults with chronic diseases questionnaire (KABQ-MS)

Xiang [27] developed the KABQ-MS in 2012 as a 40-item simplified Chinese scale that contained 3 subscales: the Knowledge, Attitude, and Behaviour of Medication Safety. First, the Knowledge of Medication Safety is a 17-item questionnaire with 5 dimensions, including basic knowledge of drugs (4 items), characteristics and principles of drug use for older adults (4 items), knowledge and treatment of adverse drug events (3 items), medication according to doctors’ advice (2 items), and drug quality identification and storage (4 items). We reverse-scored items 4, 5, 7, 9, and 13. The options were yes, no, and uncertain; the participants were given 1 point if they answered correctly and 0 points if otherwise. The total score ranged from 0 to 17. The higher the score, the greater the medication safety knowledge. Second, the Attitude Towards Medication Safety is a 10-item questionnaire with 6 dimensions, including the benefits of drug treatment (2 items), disadvantages of drug treatment (2 items), function of drug instructions (1 item), view of medication compliance (1 item), opinion of medication self-management (2 items), and attitude towards medication selection (2 items). We reverse-scored items 7, 8, 9, and 10. Responses were provided on a 5-point Likert scale ranging from 1 (strongly disagree) to 5 (strongly agree). The total score ranged from 10 to 50. The higher the score, the more positive the attitude towards safe drug use. Third, the Behaviour of Medication Safety is a 13-item questionnaire with 5 dimensions, including self-purchase drug behaviour (1 item), drug instruction reading behaviour (1 item), drug-taking behaviour (6 items), efficacy of monitoring behaviour (1 item), and drug reserving behaviour (4 items). We reverse-scored items 1, 3, 6, 7, and 8. The responses for the first 9 items ranged from never (1 point), occasionally (2 points), often (3 points), and always (4 points). The responses for the last 4 items were yes, no, and uncertain. The total score ranged from 9 to 40. The participants were given 1 point if they answered correctly and 0 points if otherwise. The higher the score, the safer the medication behaviour. It applies to a survey on knowledge, attitude, and behaviour of medication safety among community-dwelling older adults with chronic diseases [27]. The scoring rate for the knowledge, attitude, and behaviour of medication safety was determined by the average score divided by the total score multiplied by 100. A score of less than 60% was considered low, between 60 and 79% was considered medium, and above 80% was considered high. The content validity index was 0.947, the total retest reliability was 0.868, and the subscales ranged from 0.854 ~ 0.920 [27]. In this study, Cronbach’s *α* coefficient was 0.785 and the three subscales ranged from 0.676 to 0.867, indicating good reliability. We used an independent samples *t*-test and one-way analysis of variance (ANOVA) to distinguish the differences in the three subscale scores and total score according to the older adults’ demographic characteristics (discriminant validity) [28]. Discriminant validity showed that the total score was statistically significant for taking medicine kinds a day (*t* = -2.544, *p* = 0.013), education (*F* = 3.026, *p* = 0.029), residential status (*F* = 3.384, *p* = 0.018), monthly personal income (*F* = 10.305, *p* < 0.001), medical insurance type (*F* = 4.387, *p* = 0.013), taking medicine times a day (*F* = 3.596, *p* = 0.028), and whether adverse drug events have occurred (*F* = 14.958, *p* < 0.001).

### Data collection

Two postgraduate students at Fujian Medical University and a nurse at a community health service centre on Ninghua Street, Taijiang District, were the investigators during the data collection. During the preparation stage, we liaised with the supervisor of the community health service centre on Ninghua Street in advance. We emphasised the application of uniform instructions to the investigators, who underwent one or two pre-investigation training sessions. Before the data collection began, the investigators informed the participants of the study’s purpose and significance as well as their privacy rights, including their refusal to participate or answer any study questions. The participants signed an informed consent form before completing the questionnaire surveys.

During the data collection, the investigators administered the questionnaire surveys in a face-to-face manner. The investigators read every item of the questionnaire in a neutral, unbiased manner because most of the participants were aged over 60 years and had relatively low educational attainment. After ensuring that they understood the questions and answered independently, the investigators completed the questionnaires on the participants’ behalf. Upon completion, they immediately checked the questionnaires for missing answers or obvious logical errors, in which case the participants filled in or modified them on the spot. After re-checking, the investigators collected the questionnaires. The participants completed the questionnaires within 20–30 min.

After the data collection, we assigned numbers that corresponded with the participants’ names to ensure confidentiality; we also separately preserved the participants’ identities. The participants’ personal information was kept in a locked filing cabinet only accessible to the researchers. When necessary, members of government management departments or ethics committees could consult the identity information stored in the research unit according to regulations.

### Data analysis

Statistical Product Service Solutions (SPSS) version 26.0 (Armonk, New York) was used for descriptive statistics and multiple linear regression analysis. We checked continuous variables for normality prior with P-P plots and summarized scores on three dimensions (Knowledge, belief, and behaviour of medication safety) and the total score of medication safety among urban older adults by their means and standard deviations. We used multiple linear regression analysis to explore the influence of the independent variables on the dependent variable. The chosen independent variable was based on Xiang [27], who identified sociodemographic variables from interviews with older patients with chronic diseases, community healthcare workers, and experts, as well as from the prior literature and relevant questionnaires; these were used as potential variables in the multiple linear regression analysis model. Therefore, we used the participants’ demographic characteristics as the independent variables in this study. The dependent variables were the three medication safety subscales (knowledge, attitude, and behaviour) and the total score of medication safety. Multicollinearity analysis indicated that the tolerances were > 0.8 and the variance inflation factors (VIF) were < 2.0 in the four models, indicating no multicollinearity issues. Using the stepwise regression method of adding factors, we entered the independent variables into the model if the differences were statistically significant (*p*-values </ = 0.05 for the *F*-test) and excluded them if they did not statistically contribute to the prediction of the dependent variable (*p*-values >/ = 0.10 for the *F*-test). We used the standardised beta coefficient to judge the effect size of each factor and applied the coefficient of determination (*R*^*2*^) and its adjusted version to test the goodness of fit. *P* < 0.05 indicated a statistically significant difference, with 0.05 as the significance level.

## Results

### Demographics

The participants included 427 older adults with a mean age of 72.04 ± 7.45 years. Of these, 40.50% were male, 11.2 4% had attained higher education, and 75.88% were married. Regarding their reported chronic health problems, 37.00% had multiple diseases, while 43.80% had chronic illnesses for more than 10 years. Overall, 10.07% took more than five kinds of medicines a day, 26.9 3% took medicine more than three times a day, and 11.48% were hospitalised for chronic diseases (Table 1).

**Table 1:** Demographic characteristics of participants (n = 427)

Variable	Categories	*n*	%
Age, year	60–69	172	40.28
	70–79	188	44.03
	≥ 80	67	15.69
	Mean (SD)	72.04 (7.45)	
Sex	Male	173	40.50
	Female	254	59.50
Education	Primary school or below	133	31.15
	Junior high school	133	31.15
	High school or technical secondary school	113	26.46
	Junior college or above	48	11.24
Marriage	Married and spouse alive	324	75.88
	Other	103	24.12
Residential status	Live with spouse	199	46.60
	Live with children	89	20.84
	Live with spouse and children	113	26.46
	Live alone	26	6.09
The number of children	0	6	1.41
	1	195	45.67
	≥ 2	226	52.93
Occupation before retirement	Employees of enterprises and institutions	159	37.24
	Other	268	62.76
Source of income	Pension	347	81.26
	Other	80	18.74
Monthly personal income (RMB)	< 1000 yuan	48	11.24
	1000–3000 yuan	174	40.75
	3000–5000 yuan	185	43.33
	≥ 5000 yuan	20	4.68
Medical insurance type	Medical insurance for urban employees	322	75.41
	Medical insurance for urban and rural residents	97	22.72
	Other	8	1.87
Self-care	Completely self-care	395	92.51
	Partly self-care	32	7.49
	Completely unable to self-care	0	0.00
The number of chronic diseases	Single disease	269	63.00
	Coexistence of multiple diseases (≥ 2)	158	37.00
Duration of chronic disease, year	≤ 5	118	27.63
	6–10	122	28.57
	11–15	85	19.91
	16–20	59	13.82
	> 20	43	10.07
Taking medicine kinds a day	< 5	384	89.93
	≥ 5	43	10.07
Taking medicine times a day	1	176	41.22
	2	136	31.85
	≥ 3	115	26.93
Whether adverse drug events have occurred	Yes	9	2.11
	No	397	92.97
	Uncertainty	21	4.92
Whether hospitalized due to chronic diseases	Yes	49	11.48
	No	378	88.52

### Dimension and total scores of medication safety

The dimension and total scores of medication safety are shown in Table 2. The score for behaviour regarding medication safety was the highest among the three subscales, indicating that older adults’ home medication behaviour was relatively safe and standardised. The score for attitude towards medication safety was the lowest among the three subscales, indicating that older adults’ attitude towards home medication was negative. The score for medication safety knowledge was at the medium level, indicating that older adults had a basic understanding of medication safety knowledge.

**Table 2:** Dimensions scores and total score of medication safety

Dimension	Score (Mean ± SD)	Score rate (%)
Knowledge of medication safety (range of scores: 0 ~ 17)	10.77 ± 2.88	63.33
Attitude of medication safety (range of scores: 10 ~ 50)	23.69 ± 6.25	47.39
Behavior of medication safety (range of scores: 9 ~ 40)	33.81 ± 5.07	84.51
Total score (range of scores: 19 ~ 107)	68.26 ± 8.96	63.80

### Multiple linear regression analysis results

In the linear regression, as shown in Table 3, five models were generated; Model 5 had the largest predictive capacity for the total score of medication safety variability (adjusted *R*^*2*^ = 0.128; *p* < 0.05). The significant variables that negatively explained the model; that is, those that decreased the physiology and disease knowledge levels irrespective of other variables, were whether adverse drug events have occurred (*β* = −0.188; *p* < 0.001), number of chronic diseases (*β* = -0.108; *p* = 0.024), and sex (*β* = −0.091; *p* = 0.048). Meanwhile, monthly personal income (*β* = 0.243; *p* < 0.001) and taking medicine times a day (*β* = 0.173; *p* < 0.001) were associated with an increased total score of medication safety, irrespective of other variables. According to the ANOVA test, a significant linear relationship was observed between the dependent variable and set of independent variables in this model (*F* = 13.512; *p* < 0.001).

**Table 3:** Regression model: socio-demographic variables and total score

Model	*R*	*R* ^*2*^	Adjusted *R*^*2*^	Standard Error of the Estimate	ANOVA		
					*F*		Sig. *F*
1	0.260^a^	0.068	0.065	8.664	30.783		< 0.001
2	0.317^b^	0.101	0.097	8.519	23.751		< 0.001
3	0.347^c^	0.120	0.114	8.435	19.303		< 0.001
4	0.361^d^	0.130	0.122	8.398	15.798		< 0.001
5	0.372^e^	0.138	0.128	8.369	13.512		< 0.001
**Model 5**	**Unstandardised Coefficients**		**Standardised Coefficients**		**95% Confidence Interval for** ***B***		
	***B***	**Std. Error**	***Beta***	***t***	***p***	**Lower Bound**	**Upper Bound**
(Constant)	74.106	3.927	-	18.872	< 0.001	66.387	81.825
Monthly personal income	2.906	0.557	0.243	5.220	< 0.001	1.811	4.000
Whether adverse drug events have occurred	-6.392	1.563	-0.188	-4.088	< 0.001	-9.465	-3.319
Taking medicine times a day	1.901	0.527	0.173	3.606	< 0.001	0.865	2.937
The number of chronic diseases	-2.010	0.889	-0.108	-2.262	0.024	-3.756	-0.263
Sex	-1.658	0.836	-0.091	-1.982	0.048	-3.302	-0.014

*Note. R* = coefficient of determination; *F* = Fisher-Snedecor test; *β* = Regression coefficient; *t* = Student’s t-test; a = Monthly personal income; b = Monthly personal income, whether adverse drug events have occurred; c = Monthly personal income, whether adverse drug events have occurred, taking medicine times a day; d = Monthly personal income, whether adverse drug events have occurred, taking medicine times a day, the number of chronic diseases; e = Monthly personal income, whether adverse drug events have occurred, taking medicine times a day, the number of chronic diseases, sex. Correlation is significant at the 0.05 level

As shown in Table 4, three models were generated; Model 3 had the largest predictive capacity for the knowledge score of medication safety variability (adjusted *R*^*2*^ = 0.062; *p* < 0.05). The significant variables that negatively explained the model; that is, those that decreased the physiology and disease knowledge levels irrespective of other variables, were whether adverse drug events have occurred (*β* = -0.166; *p* = 0.001) and the number of chronic diseases (*β* = -0.166; *p* = 0.001). Meanwhile, education (*β* = 0.123; *p* = 0.009) was associated with an increased knowledge score of medication safety, irrespective of other variables. According to the ANOVA test, a significant linear relationship was observed between the dependent variable and set of independent variables in this model (*F* = 10.356; *p* < 0.001).

**Table 4:** Regression model: socio-demographic variables and knowledge of medication safety

Model	*R*	*R* ^*2*^	Adjusted *R*^*2*^	Standard Error of the Estimate	ANOVA		
					*F*		Sig. *F*
1	0.164^a^	0.027	0.025	2.846	11.773		0.001
2	0.231^b^	0.053	0.049	2.811	11.972		< 0.001
3	0.262^c^	0.068	0.062	2.792	10.356		< 0.001
**Model 3**	**Unstandardised Coefficients**		**Standardised Coefficients**		**95% Confidence Interval for** ***B***		
	***B***	**Std. Error**	***Beta***	***t***	***p***	**Lower Bound**	**Upper Bound**
(Constant)	14.872	1.184	-	12.558	< 0.001	12.544	17.200
Whether adverse drug events have occurred	-1.755	0.514	-0.161	-3.415	0.001	-2.765	-0.745
The number of chronic diseases	-0.962	0.280	-0.161	-3.437	0.001	-1.512	-0.412
Education	0.354	0.136	0.123	2.607	0.009	0.087	0.621

*Note. R* = coefficient of determination; *F* = Fisher-Snedecor test; *β* = Regression coefficient; *t* = Student’s t-test; a = Whether adverse drug events have occurred; b = Whether adverse drug events have occurred, the number of chronic diseases; c = Whether adverse drug events have occurred, the number of chronic diseases, education. Correlation is significant at the 0.05 level

As shown in Table 5, four models were generated; Model 4 had the largest predictive capacity for the attitude score of medication safety variability (adjusted *R*^*2*^ = 0.066; *p* < 0.05). The significant variables that negatively explained the model; that is, those that decreased the physiology and disease knowledge levels irrespective of other variables, were whether adverse drug events have occurred (*β* = -0.132; *p* = 0.005) and the number of chronic diseases (*β* = -0.099; *p* = 0.050). Meanwhile, taking medicine kinds a day (*β* = 0.167; *p* = 0.001) and taking medicines times a day (*β* = 0.154; *p* = 0.003) were associated with increased attitude scores of medication safety, irrespective of other variables. According to the ANOVA test, a significant linear relationship was observed between the dependent variable and set of independent variables in this model (*F* = 8.558; *p* < 0.001).

**Table 5:** Regression model: socio-demographic variables and attitude of medication safety

Model	*R*	*R* ^*2*^	Adjusted *R*^*2*^	Standard Error of the Estimate	ANOVA		
					*F*		Sig. *F*
1	0.190^a^	0.036	0.034	6.146	15.855		< 0.001
2	0.228^b^	0.052	0.048	6.102	11.667		< 0.001
3	0.258^c^	0.067	0.060	6.062	10.052		< 0.001
4	0.274^d^	0.075	0.066	6.042	8.558		< 0.001
**Model 4**	**Unstandardised Coefficients**		**Standardised Coefficients**		**95% Confidence Interval for** ***B***		
	***B***	**Std. Error**	***Beta***	***t***	***p***	**Lower Bound**	**Upper Bound**
(Constant)	25.799	2.569	-	10.041	< 0.001	20.749	30.850
Taking medicine kinds a day	3.462	1.054	0.167	3.286	0.001	1.391	5.534
Whether adverse drug events have occurred	-3.133	1.111	-0.132	-2.821	0.005	-5.316	-0.950
Taking medicine times a day	1.181	0.396	0.154	2.980	0.003	0.402	1.961
The number of chronic diseases	-1.283	0.652	-0.099	-1.967	0.050	-2.564	-0.001

*Note. R* = coefficient of determination; *F* = Fisher-Snedecor test; *β* = Regression coefficient; *t* = Student’s t-test; a = Taking medicine kinds a day; b = Taking medicine kinds a day, whether adverse drug events have occurred; c = Taking medicine kinds a day, whether adverse drug events have occurred, taking medicine times a day; d = Taking medicine kinds a day, whether adverse drug events have occurred, taking medicine times a day, the number of chronic diseases. Correlation is significant at the 0.05 level

As shown in Table 6, five models were generated; Model 5 had the largest predictive capacity for the behaviour score of medication safety variability (adjusted *R*^*2*^ = 0.222; *p* < 0.05). The significant variables that negatively explained the model; that is, those that decreased the physiology and disease knowledge levels irrespective of other variables, were self-care (*β* = -0.160; *p* < 0.001), hospitalisation due to chronic diseases (*β* = −0.152; *p* = 0.001), and sex (*β* = -0.103; *p* = 0.018). Meanwhile, monthly personal income (*β* = 0.375; *p* < 0.001) and taking medicine times a day (*β* = 0.096; *p* = 0.026) were associated with increased behaviour scores of medication safety, irrespective of other variables. According to the ANOVA test, a significant linear relationship was observed between the dependent variable and set of independent variables in this model (*F* = 25.275; *p* < 0.001).

**Table 6:** Regression model: socio-demographic variables and behavior of medication safety

Model	*R*	*R* ^*2*^	Adjusted *R*^*2*^	Standard Error of the Estimate	ANOVA		
					*F*		Sig. *F*
1	0.384^a^	0.148	0.146	4.688	73.559		< 0.001
2	0.435^b^	0.189	0.185	4.577	49.492		< 0.001
3	0.459^c^	0.211	0.205	4.522	37.605		< 0.001
4	0.471^d^	0.222	0.214	4.495	30.052		< 0.001
5	0.480^e^	0.231	0.222	4.474	25.275		< 0.001
**Model 5**	**Unstandardised Coefficients**		**Standardised Coefficients**		**95% Confidence Interval for** ***B***		
	***B***	**Std. Error**	***Beta***	***t***	***p***	**Lower Bound**	**Upper Bound**
(Constant)	30.531	1.277	-	23.899	< 0.001	28.020	33.042
Monthly personal income	2.536	0.296	0.375	8.581	< 0.001	1.955	3.117
Self-care	-3.072	0.858	-0.160	-3.580	< 0.001	-4.759	-1.385
Whether hospitalized due to chronic diseases	-1.812	0.535	-0.152	-3.383	0.001	-2.864	-0.759
Sex	-1.067	0.451	-0.103	-2.369	0.018	-1.953	-0.182
Taking medicine times a day	0.600	0.268	0.096	2.241	0.026	0.074	1.127

*Note. R* = coefficient of determination; *F* = Fisher-Snedecor test; *β* = Regression coefficient; *t* = Student’s t-test; a = Monthly personal income; b = Monthly personal income, self-care; c = Monthly personal income, self-care, whether hospitalized due to chronic diseases; d = Monthly personal income, self-care, whether hospitalized due to chronic diseases, sex; e = Monthly personal income, self-care, whether hospitalized due to chronic diseases, sex, taking medicine times a day. Correlation is significant at the 0.05 level

## Discussion

### Home-based medication safety among older adults with chronic diseases

The average score of home-based medication safety among older adults with chronic diseases was 68.26 ± 8.96, and the total score was 63.80%, indicating that community-dwelling older patients had a moderate grasp of medication safety. However, older adults had uneven scoring in all aspects of home-based medication safety. The scoring rate of each subscale ranked from high to low was behaviour (84.51%), knowledge (63.33%), and attitude (47.39%). Community-dwelling older adults with chronic diseases had a low grasp of medication safety knowledge and a moderate level of medication safety beliefs. Although the scoring rate of medication safety behaviour is high, some patients still have safety risks in medication behaviour, which is harmful or even fatal for individuals.

### Knowledge of home-based medication safety among older adults with chronic diseases

Correct knowledge is the basis for building positive beliefs and the precondition for forming behaviours [29]. Most studies [10, 30–32] have found that older adults lack knowledge of home-based medication safety, while drug identification, pharmacological actions, and storage are of particular concern. Neoh et al. [33] investigate 79 community-dwelling older adults in Malaysia and reveal that approximately 75.0% are unable to distinguish between genuine and fake drugs. Xu et al. [10] investigate 400 community-dwelling older adults with chronic diseases in Beijing and find that their scores for identifying verified and fake drugs (0.38 ± 0.91) and understanding drug interactions (0.72 ± 1.11) are far lower than the median of 3 points. Zhang et al. [34] investigate the home-based drug knowledge of Uyghur and Han older adults and state that 76.7% of Uyghurs and 66.7% of Han people do not understand drug incompatibility, while 86.0% of Uyghurs and 66.7% of Han people have little knowledge of drug storage. Therefore, community-dwelling older adults have a low level of knowledge about home-based medication safety, and their cognitive abilities in this regard must be improved.

Older adults mostly acquire their home-based medication safety knowledge from medical personnel. Lin et al. [35] investigate 283 older adults in communities in Guangzhou and reveal that 72.1% of their drug use information is from doctors in hospitals, while 12.7% is from pharmacists. Xu et al. [10] also reveal that older adults primarily acquire their medication knowledge from medical staff (308 doctors, 77.0%; 107 nurses, 26.8%) and healthcare programmes on television (220 people, 55.0%). When older adults have questions about their medication, more than 60% will consult medical personnel [36]. Therefore, the medical community should make greater efforts to improve older adults’ awareness of medication safety at home. For example, healthcare workers could establish more health education programmes to enrich knowledge about safe medication use, conduct more health records and follow-ups to guide older adults on medication safety, and provide drug counselling services in the community to facilitate older adults’ understanding of drug-use information [11].

### Attitude towards home-based medication safety among older adults with chronic diseases

Beliefs and attitudes are the driving forces that change behaviours. People transform their knowledge into beliefs and attitudes by thinking independently, which further dominates their behaviour [29]. Although most older adults believe that taking medication is significant for maintaining their current and future health [33], their medication safety beliefs are at a low level [10, 30, 31], which is consistent with our study results. In China, most older adults think that traditional Chinese medicine is safe and has no toxic or side effects, so taking it to recuperate their bodies is a casual practice [30]. Most older adults abroad believe that over-the-counter drugs are safe, so when they want to quickly relieve their illness, they will take over-the-counter drugs that exceed the recommended dosage or frequency [37, 38]. Jiang et al. [30] find that 72.9% of 210 community-dwelling older adults believe that that both distributing drugs to each other within their circle of family and friends and forgetting to take drugs are acceptable; meanwhile, 13.3% believe that the more drugs and the greater the dose, the better the efficacy. Furthermore, older adults both in China and abroad believe that they may adjust their daily medication based on their experiences and feelings [10, 37, 38]. According to the theory of knowledge, beliefs, and practice [20], rich and correct knowledge forms the basis of healthy beliefs. Therefore, to change older adults’ attitudes and potentially adverse behaviours regarding medication safety, it is necessary to continue to both enrich their knowledge and change their negative attitudes towards medication safety.

### Behaviour of home-based medication safety among older adults with chronic diseases

Behaviour constitutes the practice of knowledge that people believe and have mastered. The more knowledge and positive attitudes people have, the more conducive they will be to the formation of effective behaviour [29]. Community-dwelling older adults have three main problems regarding their home-based medication safety behaviour. First, in terms of drug storage, Gao et al. [39] find that in the two new and old communities in Shanghai, the rate of home medicine kits for empty-nesters is low, accounting for 33.3% and 19.0%, respectively. Moreover, most store drugs in drawers and some even place them at will. Lin et al. [35] find that among 283 older adults in Guangzhou communities, 44.9% place medicines at home at will, while 50% do not regularly clean and store medicines [39]. This will increase the incidence of adverse drug events, such as taking drugs that have expired by mistake. Second, in terms of drug compliance, Chew et al. [40] state that only 40.0% of 400 community-dwelling older adults with chronic diseases in Singapore insist on taking drugs, and that the noncompliance rate is as high as 60.0%. Qiao et al. [41] investigate 780 community-dwelling older adults with chronic diseases from Jinan and reveal that 381 have poor drug compliance. However, other studies [10, 35, 42] have shown that more than 60.0% of older adults have good drug compliance. For example, Lin et al. [35] survey 283 community-dwelling older adults in Guangzhou and reveal that 72.4% take medicine according to doctors’ advice. This difference may be related to the differences in medical service security systems in various regions. Third, drug abuse is common. Odani et al. [43] analyse drug-use data from 2015 to 2017 in the United States and reveal that approximately 4 million community-dwelling older adults abuse medication, of which 37.9% comprises of painkillers. Shah et al. [36] state that most older adults will continue to choose a drug because of its past effectiveness and comfort, even though it may be unsuitable for current symptoms. Therefore, it is important to cultivate older adults’ medication safety behaviours. Regional medical security systems should be improved, and medical personnel should strengthen their medication safety education to correct older adults’ incorrect drug-taking concepts and form safe drug-taking behaviours.

### Influencing factors of home-based medication safety among older adults with chronic diseases

The influencing factors of home-based medication safety among older adults with chronic diseases include monthly personal income, adverse drug events, taking medicine times a day, the number of chronic diseases, sex, education, taking medicine kinds a day, self-care, and hospitalisation due to chronic diseases. First, the higher the monthly personal income, the safer the medication behaviour, which is consistent with the results of Milan et al. [44] and Dillon et al. [45]. Older patients’ compliance will gradually decrease with increased self-paid drug costs. Second, the occurrence of adverse drug events influences older adults’ knowledge and attitude towards medication safety. Due to lack of knowledge, older adults have poor drug management, inducing adverse drug events. By involving older adults as active partners in their healthcare, many errors and medication-related health problems can be prevented [46]. Third, the higher the frequency of taking medication a day, the more positive the attitude and safer the behaviour towards drug use. A possible reason for this is because during our investigation, we found that some older adults reported that the more they took medication a day, the more their familiarity with drugs, and the more their medication safety improved. Fourth, the lower the number of chronic diseases, the higher the knowledge of medication safety, and the more positive the attitude towards drug use. This is consistent with the results of Zhang et al. [13], who find that among 412 older patients with chronic diseases in Shanghai, the higher the number of diseases, the lower the medication compliance, and the more prone they will be to medication-related problems. Fifth, sex influences older adults’ medication safety behaviour. Among older adults, females are at the greatest risk of drug-related harm [47]; therefore, they pay more attention to medication safety. Sixth, the greater their educational attainment, the more channels that people have to obtain knowledge; thus, education influences older adults’ medication safety knowledge [32], which is consistent with our results. The higher the educational level of older adults, the better their ability to receive and understand information, and the higher their awareness of medication safety [48]. Seventh, the more kinds of drugs that are used a day, the more positive the attitudes towards drug use. This appears to be inconsistent with the finding that older adults’ non-compliance rate will increase by 1.16 times [44] for every additional drug used. We believe that there may be two reasons for this inconsistency. First, our data showed that 384 of the 427 older adults (89.93%) took less than five kinds of drugs a day, while the proportion of older adults who took more than five kinds of drugs was very small (10.07%); this may have affected the analysis results. Second, some older adults said that although they took multiple drugs a day, they had formed the habit of actively doing so to control their diseases. Eighth, the better the self-care ability, the safer the medication behaviour, which is consistent with the results of Zhao et al. [49], who reveal that self-care can improve the safety and compliance of medications in patients with chronic heart failure. Therefore, patients can actively improve their self-care ability to promote their health. Finally, non-hospitalised older adults with chronic diseases have safer drug-use behaviours than hospitalised patients. Hospitalised older adults may have multiple illnesses and take more complex drugs [50]. Moreover, hospitalisation may introduce medication changes [51] combined with the inadequate transfer of medication-related information at discharge; this may lead to unsafe medication practices at home, which may have a greater impact on medication safety [52]. Therefore, hospitalised patients face more potential safety hazards in their drug-use behaviours compared to non-hospitalised patients.

In short, community-dwelling older adults with chronic diseases had a low grasp of medication safety knowledge, and their knowledge reserve needed to be improved. Their belief in drug safety was medium, so they should set up more correct and active drug safety concepts. Simultaneously, we should take personalized corrective measures in the face of individuals’ drug safety misconduct. It is necessary to give full play to the advantages of various healthcare interventions in the community, make full use of medical resources, and ensure that older adults receive comprehensive and timely medication-related guidance. Moreover, medical staff and community workers should pay attention to the drug safety of older adults with different characteristics and mobilize their enthusiasm for participation to improve their medication self-management ability.

## Limitations

This study had the following limitations. First, it was limited by its cross-sectional design, which could not infer causality among the variables or account for unknown confounders. Second, although we included as many influencing factors of home-based medication safety as possible, other related factors were not explored, such as the type of chronic disease. Finally, we only surveyed older adults in urban communities in Fuzhou due to time, personnel, and material resource limitations. Thus, the generalisability of our results may be limited. The future research should consider expanding the scope to different cities and conducting multicentre large-sample surveys to explore and compare the knowledge, attitude, and behaviour of medication safety among older adults with chronic diseases using random stratified sampling.

## Electronic supplementary material

Below is the link to the electronic supplementary material.

Supplementary Material 1. Participants’ Demographic Characteristics Questionnair

## Data Availability

The datasets generated and/or analyzed during the current study are not publicly available due this article is part of the author’s master’s thesis with a confidentiality period of 2 years, and other related papers have not yet been published. The datasets were not suitable for publication now but are available from the corresponding author upon reasonable request.

## Abbreviations

ANOVA: One-way analysis of variance
KABQ-MS: Knowledge, Attitude, and Behaviour of Medication Safety among Older Adults with Chronic Diseases Questionnaire
VIF: Variance inflation factors
