# Supplementary material for: Knowledge, attitude, behaviour, and influencing factors of home-based medication safety among community-dwelling older adults with chronic diseases: a cross-sectional study
Source: BMC Geriatr. 2023 Apr 28;23:256. doi: 10.1186/s12877-023-03966-3 (PMC10148421; doi:10.1186/s12877-023-03966-3)
Supplement: Supplementary file 1 — Supplementary Material 1. Participants’ Demographic Characteristics Questionnair [file 12877_2023_3966_MOESM1_ESM.docx]

**Knowledge, attitude, behaviour, and influencing factors of home-based** **medication safety among** **community-dwelling older adults with chronic diseases: A cross-sectional study**

**Participants’ Demographic Characteristics Questionnaire**

**1.** **Sex:** A. Male B. Female

**2.** **Age:**  years old

**3.** **Education:**

A. Primary school or below

B. Junior high school

C. High school or technical secondary school

D. Junior college or above

**4.** **Marriage:**

A. Married and spouse alive

B. Married and spouse absent

C. Divorced

D. Single

**5.** **Residential status:**

A. Live with spouse

B. Live with children

C. Live with spouse and children

D. Live alone

**6.** **The number of children****:**

**7. Occupation before retirement (single or multiple choice available):**

A. Peasants

B. Workers

C. Employees of institutions

D. Employees of state-owned enterprises

E. Employees of private enterprises

F. Medical, educational and scientific workers

G. Self-employed person

H. Freelancers

I. Other _________________

**8.** **Source of income (single or multiple choice available):**

A. Pension

B. Income from current employment

C. Children support payments

D. Other _________________

**9. Monthly personal income (RMB):**

A. Less than1000 yuan

B. 1000-3000 yuan

C. 3000-5000 yuan

D. 5000 yuan and above

**10.** **Medical insurance type:**

A. Medical insurance for urban employees

B. Medical insurance for urban and rural residents

C. New Rural Cooperative Medical Care

D. Commercial Insurance

E. Not insured

F. Other _________________

**11.** **How well do you feel able to take care of yourself in life (single choice):**

A. Completely self-care

B. Partly self-care

C. Completely unable to self-care

**12.** **The type of chronic disease you currently have and duration you have had it (multiple choice available):**

A. Hypertension year(s)

B. Diabetes year(s)

C. Hyperlipidemia year(s)

D. Coronary heart disease year(s)

E. Chronic kidney disease year(s)

F. Chronic Obstructive Pulmonary Disease year(s)

G. Asthma year(s)

H. Gout year(s)

I. Rheumatoid arthritis year(s) 7

J. Cirrhosis of the liver year(s)

K. Peptic ulcer year(s) 7

L. Other year(s)

**13.** **How many times have you been hospitalized in the last year due to a chronic illness you have suffered from？** time(s)

**14.** **The number of medications you take a day is:**

A. two types and below

B. three to five types

C. six to nine types

D. ten types and above

**15.** **The number of times you take your medication a day is:**

A. once

B. twice

C. three times and above

**16.** **Have you had any adverse drug events to your medication in the last three months:**

A. Yes（Please describe it ）

B. No

C. Uncertainty

**Knowledge of Medication Safety among Older Adults with Chronic Diseases Questionnaire**

Instructions: Please mark "√" in the column you think is appropriate.

**1. Medicines are divided into prescription and over-the-counter medicines (OTC medicines).**

A. Yes B. No C. Uncertainty

**2. Drugs have both therapeutic and toxic side effects.**

A. Yes B. No C. Uncertainty

**3. Chinese medicines (or proprietary Chinese medicines) can also have toxic side effects.**

A. Yes B. No C. Uncertainty

**4. Tonic medicine is good for the body and not harmful.**

A. Yes B. No C. Uncertainty

**5. The more types of medication you use, the more effective your treatment will be.**

A. Yes B. No C. Uncertainty

**6. The dose of medication for older adults is smaller than that for the average adult.**

A. Yes B. No C. Uncertainty

**7. Older adults are less likely to experience adverse reactions to medication than the average adult.**

A. Yes B. No C. Uncertainty

**8. Medication for older adults needs to take into account the function of the liver and kidneys.**

A. Yes B. No C. Uncertainty

**9. The type and dosage of medication should be the same for different people with the same disease.**

A. Yes B. No C. Uncertainty

**10. The more types and doses of drugs used, the more likely it is that adverse drug reactions will occur.**

A. Yes B. No C. Uncertainty

**11. The drug should be discontinued immediately if adverse reactions occur during administration.**

A. Yes B. No C. Uncertainty

**12. I should take my medication exactly as prescribed by my doctor.**

A. Yes B. No C. Uncertainty

**13. You can stop taking the medication when you are well and have no discomfort.**

A. Yes B. No C. Uncertainty

**14. I know how to judge the quality of medicines.**

A. Yes B. No C. Uncertainty

**15. Do not take medicines after they have changed color or become damp.**

A. Yes B. No C. Uncertainty

**16. Taking expired medicines can be harmful to your health.**

A. Yes B. No C. Uncertainty

**17. Store medicines in accordance with the prescribed storage conditions.**

A. Yes B. No C. Uncertainty

**Attitude of Medication Safety among Older Adults with Chronic Diseases Questionnaire**

Instructions: Please mark "√" in the column you think is appropriate.

**1. Medication can ease the pain and make my life better.**

A. Strongly agree B. Agree C. Uncertainty D. Disagree E. Strongly disagree

**2. The benefits of drugs generally outweigh the harms.**

A. Strongly agree B. Agree C. Uncertainty D. Disagree E. Strongly disagree

**3. Worried that long-term medication may be harmful to your health.**

A. Strongly agree B. Agree C. Uncertainty D. Disagree E. Strongly disagree

**4. Fear that long-term medication will lead to dependency.**

A. Strongly agree B. Agree C. Uncertainty D. Disagree E. Strongly disagree

**5. Drug instructions are a guide to taking medication.**

A. Strongly agree B. Agree C. Uncertainty D. Disagree E. Strongly disagree

**6. The doctor's advice to me has merit, so it should be followed.**

A. Strongly agree B. Agree C. Uncertainty D. Disagree E. Strongly disagree

**7. You can manage your daily medication with your own experience and feeling.**

A. Strongly agree B. Agree C. Uncertainty D. Disagree E. Strongly disagree

**8. You can manage your daily medication through advertising and referrals from friends.**

A. Strongly agree B. Agree C. Uncertainty D. Disagree E. Strongly disagree

**9. Expensive, new drugs are more effective than cheaper, older drugs.**

A. Strongly agree B. Agree C. Uncertainty D. Disagree E. Strongly disagree

**10. Prescriptions and recipes are more effective than ordinary medicines.**

A. Strongly agree B. Agree C. Uncertainty D. Disagree E. Strongly disagree

**Behaviour of Medication Safety among Older Adults with Chronic Diseases Questionnaire**

Instructions: Please mark "√" before the serial number you think is appropriate.

**1.** **In the absence of a doctor's diagnosis, will you go to the pharmacy to buy medicines on your own?**

A. Never B. Occasionally C. Often D. Always

**2.** **Do you read the medication instructions before administering the medicine?**

A. Never B. Occasionally C. Often D. Always

**3.** **Have you stopped taking your medication on your own after your condition has improved without your doctor's consent?**

A. Never B. Occasionally C. Often D. Always

**4.** **Can you take your medication as often as your doctor wants you to every day?**

A. Never B. Occasionally C. Often D. Always

**5.** **Can you take your medication every day as prescribed by your doctor?**

A. Never B. Occasionally C. Often D. Always

**6.** **Have you changed the dosage of your medication without your doctor's consent?**

A. Never B. Occasionally C. Often D. Always

**7.** **Have you changed the type of medication you take without your doctor's consent?**

A. Never B. Occasionally C. Often D. Always

**8.** **Did you use the wrong type or dose of medication?**

A. Never B. Occasionally C. Often D. Always

**9.** **Can you have regular blood pressure, blood sugar and other routine tests to see how well your medication is working?**

A. Never B. Occasionally C. Often D. Always

**10.** **Do you have a special medicine cabinet (or cabinet) at home where you can store your medicines？**

A. Yes B. No C. Uncertainty

**11.** **Do you store your internal and external medicines separately?**

A. Yes B. No C. Uncertainty

**12.** **Do you regularly check the expiry date and quality of your medicines?**

A. Yes B. No C. Uncertainty

**13.** **Do you discard expired or spoiled medicines?**

A. Yes B. No C. Uncertainty
